# Molecular Biology and Infection of Hepatitis E Virus

**DOI:** 10.3389/fmicb.2016.01419

**Published:** 2016-09-07

**Authors:** Yuchen Nan, Yan-Jin Zhang

**Affiliations:** ^1^Department of Preventive Veterinary Medicine, College of Veterinary Medicine, Northwest A&F UniversityYangling, China; ^2^Molecular Virology Laboratory, VA-MD College of Veterinary Medicine and Maryland Pathogen Research Institute, University of Maryland, College Park, College ParkMD, USA

**Keywords:** hepatitis E virus, HEV, HEV biology, viral proteins of HEV, HEV infection, HEV vaccine

## Abstract

Hepatitis E virus (HEV) is a viral pathogen transmitted primarily via fecal-oral route. In humans, HEV mainly causes acute hepatitis and is responsible for large outbreaks of hepatitis across the world. The case fatality rate of HEV-induced hepatitis ranges from 0.5 to 3% in young adults and up to 30% in infected pregnant women. HEV strains infecting humans are classified into four genotypes. HEV strains from genotypes 3 and 4 are zoonotic, whereas those from genotypes 1 and 2 have no known animal reservoirs. Recently, notable progress has been accomplished for better understanding of HEV biology and infection, such as chronic HEV infection, *in vitro* cell culture system, quasi-enveloped HEV virions, functions of the HEV proteins, mechanism of HEV antagonizing host innate immunity, HEV pathogenesis and vaccine development. However, further investigation on the cross-species HEV infection, host tropism, vaccine efficacy, and HEV-specific antiviral strategy is still needed. This review mainly focuses on molecular biology and infection of HEV and offers perspective new insight of this enigmatic virus.

## Introduction

Hepatitis E virus (HEV) is a positive-sense, single-stranded RNA virus, and is classified in the genus *Orthohepevirus*, the family *Hepeviridae* (Smith et al., 2014). The HEV-caused hepatitis E is generally a self-limiting disease with a case fatality rate from 0.5 to 3% in young adults but up to 30% in infected pregnant women in their third trimester of gestation (Jameel, 1999; Nan, 2014). World Health Organization (WHO) estimates that there are 20 million infections with over 3 million symptomatic cases and 56,600 deaths annually across the world (WHO, 2015). HEV is primarily transmitted via fecal-oral route. HEV infection was previously thought to be a public health problem only for the developing countries. Indeed, hepatitis E is highly endemic in East and South Asia, as well as Africa according to the WHO (WHO, 2015). HEV strains infecting humans are classified into four genotypes. HEV strains from genotypes 3 and 4 are zoonotic, whereas, those from genotypes 1 and 2 have no known animal origin. Discovery of HEV from swine and other species suggests that genotypes 3 and 4 HEV has a wide host range (Christensen et al., 2008; Dalton et al., 2008; Meng, 2013; Pavio et al., 2015). Currently, hepatitis E is frequently recognized in industrialized countries, where it was not thought to be endemic (Kwo et al., 1997; Erker et al., 1999; Schlauder et al., 1999; Worm et al., 2000; Kabrane-Lazizi et al., 2001; Mizuo et al., 2002; Sadler et al., 2006). Moreover, along with isolation of HEV from the pig, chicken, mongoose, rabbit, rat, ferret, bat, fish, and deer (Meng et al., 1997; Haqshenas et al., 2001; Li et al., 2005c; Cossaboom et al., 2012; Smith et al., 2014), cross-species infection of HEV from animal reservoirs to humans is thought to be the major cause of sporadic cases of hepatitis E in the industrialized countries (Pavio et al., 2015). Although previously thought to only cause acute infections, HEV is found in chronic infections reported both in immune compromised and immunocompetent individuals (Hoofnagle et al., 2012; Grewal et al., 2014). In addition, extrahepatic manifestations, such as neurological disorders and kidney injury in HEV infected patients have been documented (Kamar et al., 2011, 2012b; van Eijk et al., 2014; Dalton et al., 2016; Geng et al., 2016). Taken together, current knowledge for HEV implies a significant underestimation of HEV infection as a public health concern. In the following sections, recent progress in HEV biology, functions of viral proteins, cell culture system, epidemiology, viral pathogenesis, treatment, and vaccine development are reviewed and perspective new insights are discussed.

## HEV Biology

Hepatitis E was initially designated as enterically transmitted non-A, non-B hepatitis (ET-NANBH) due to similar clinical presentations to hepatitis A and B in patients, but the prospective causative agent was initially unknown (Balayan et al., 1983). Early research implied that an RNA virus was the potential pathogen for the ET-NANBH. By analysis of a cDNA library from infectious bile sample, a portion of a highly conserved RNA-dependent RNA-polymerase (RdRp) motif, commonly found in RNA viruses, was identified (Reyes et al., 1990). This new virus was designated as HEV, which was responsible for the outbreak of ET-NANBH.

**FIGURE 1:**
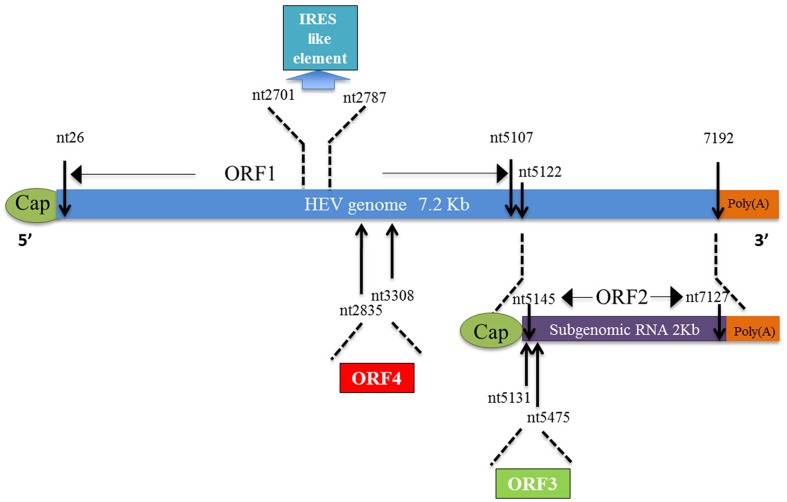
**Schematic illustration of hepatitis E virus (HEV) genome, subgenomic RNA, and ORFs.** ORF1 (nt 26–5107) is labeled above the genomic RNA box. ORF2 (nt 5145–7127) and ORF3 (nt 5131–5475) are encoded by the same subgenomic RNA. The newly identified ISRE like sequence (nt 2701–2787) and ORF4 (nt 2835–3308) which are overlapped with ORF1 are listed as well. Moreover, the numbers above or below the RNA boxes indicate nucleotide numbers of the cDNA of HEV Sar55 (GenBank accession number AF444002) genomic RNA.

The complete sequence of HEV genome was published 1 year later (Tam et al., 1991). Sequence analysis indicated that HEV contains a 7.2 kb single-stranded positive-sense RNA genome, which is capped and poly-adenylated (Ahmad et al., 2011). There are three partially overlapped open reading frames (ORFs) in an order of sequences encoding non-structural proteins (NSPs) followed by structural protein (Tam et al., 1991; Tsarev et al., 1992; **Figure 1**). HEV ORF1 encodes a non-structural polyprotein that consists of replicase proteins needed for HEV replication. ORF2 encodes the capsid protein, which is the major structural protein of the HEV virions, which are non-enveloped particles of 32–34 nm in diameter (Mori and Matsuura, 2011). ORF3 encodes a small multifunctional protein with a molecular mass of 13 kDa (VP13). There are also short untranslated regions (UTRs) in both the 5′ and 3′-end of the genome. Recently, an ORF4 was identified from genotype 1 HEV solely (Nair et al., 2016; **Figure 1**). Expression of ORF4 is cap-independent and driven by a putative IRES-like element between 2701 and 2787 nt of the HEV genome (Nair et al., 2016).

The ORF1 of HEV can be translated directly from the genomic RNA, whereas ORF2 and ORF3 are translated soly from the sub-genomic RNA in alternative frames (Graff et al., 2006). In an earlier report, three RNA species were detected in liver tissue of experimentally infected macaques, with sizes of 7.2, 3.7, and 2 kb (Tam et al., 1991). The 3.7 and 2 kb RNA species were thought to be sub-genomic RNAs for translation of ORF2 and ORF3, respectively. However, a later study in Huh7 cells only identified one capped 2.2 kb sub-genomic RNA, which is a bicistronic mRNA for translation of both ORF2 and ORF3 (Graff et al., 2006). Transcription of this sub-genomic RNA initiates at nucleotide position 5122 in the Sar55 strain, which is located downstream of the first two methionine codons of the initially presumed ORF3. The same conclusion was drawn from another *in vitro* study of genotype 3 HEV infection in PLC/PRF/5 hepatoma cells (Ichiyama et al., 2009). The HEV genome contains two *cis*-reactive elements (CREs) that are essential for the viral replication (Cao et al., 2010; Parvez, 2015b). The first CRE overlaps the 3′ end of ORF2 and the 3′ UTR and is essential for HEV replication. The second CRE locates in the intergenic region of the HEV genome and forms a stem–loop structure that may be the promoter for synthesis of the 2.2-kb subgenomic RNA (Cao et al., 2010).

## Genotypes of HEV Strains

Hepatitis E virus was initially classified as a member of the *Caliciviridae* family. However, sequence analysis of HEV ORF1 indicated no similarity to Caliciviruses, or other picorna-like viruses. On the other hand, there is limited but significant similarity to the alphavirus-like superfamily of RNA viruses, specifically, the rubella virus (Berke and Matson, 2000). Consequently, HEV was classified into the family *Hepeviridae* (Berke and Matson, 2000; Emerson and Purcell, 2003).

Although HEV strains are highly diverse and heterogenic, only one serotype of HEV exists. Classification of HEV strains is under transition due to the different criteria used (Smith et al., 2013, 2014). Recently, a new proposal for the classification of the family *Hepeviridae* was published (Smith et al., 2014). In this proposal, the family *Hepeviridae* contains two genera: *Orthohepevirus* (all mammalian and avian HEV isolates) and *Piscihepevirus* (trout HEV; **Table 1**). Within the genus *Orthohepevirus*, four different species (A, B, C, D) are designated to include isolates from different hosts (Smith et al., 2014). All four previously recognized HEV genotypes (1–4) that infect humans belong to the *Orthohepevirus A* virus (Smith et al., 2014).

**Table 1:** Hepatitis E virus (HEV) genotypes, natural hosts, and zoonotic infection to humans.

Genus	Species	Genotype	Natural hosts	Infection to humans
*Orthohepevirus*	*Orthohepevirus A*	1	Human	Yes
		2	Human	Yes
		3	Human, pig, rabbit, deer, mongoose, wild boar	Yes
		4	Human, pig, yak, wild boar	Yes
		5	Wild boar	Unknown
		6	Wild boar	Unknown
		7	Camel	Yes
	*Orthohepevirus B*		Chicken	No
	*Orthohepevirus C*	C1	Rat	No
		C2	Ferret	No
	*Orthohepevirus D*		Bat	No
*Piscihepevirus*	*Piscihepevirus A*		Trout	No


The previously recognized HEV genotypes 1–4 classification system was based on complete genomic sequences (Lu et al., 2006). HEV genotype 1 is the most conserved among the four genotypes. There is only one full-length genotype 2 sequence available (Smith et al., 2016). Both HEV genotypes 1 and 2 are restricted to humans with no known animal reservoirs, whereas genotypes 3 and 4 are zoonotic with an expanded host range (**Table 1**; Meng, 2010; Ahmad et al., 2011). Therefore, genotypes 3 and 4 HEV strains are highly diverse (Lu et al., 2006; Smith et al., 2014). Since the constant discovery of new HEV or HEV-related isolates from rabbit, rat, ferret, bat, moose, farmed mink, camel, and wild boar (Lorenzo et al., 2007; Zhao et al., 2009; Johne et al., 2010; Geng et al., 2011; Takahashi et al., 2011; Drexler et al., 2012; Raj et al., 2012; Krog et al., 2013; Lin et al., 2014; Woo et al., 2014), four genotypes are no longer satisfying classification of expanding HEV isolates. In the new classification system, some HEV strains from wild boars in Japan with unique viral nucleotide sequences are designated as genotypes 5 and 6, while HEV from camel is classified as genotype 7 of *Orthohepevirus A* (Smith et al., 2014). The genotypes that infect humans include 1, 2, 3, 4, and 7 (**Table 1**; Smith et al., 2016).

Hepatitis E virus-like virus isolated from avian species is called avian HEV, which shares less than 50% nucleotide identity but common antigen epitopes in the capsid protein with mammalian HEV (Haqshenas et al., 2001, 2002; Huang et al., 2004). Currently, avian HEV is classified into the species *Orthohepevirus B* (Smith et al., 2014). HEV strains from rat, ferret and bat are classified into the species of *Orthohepevirus C* and *D*, respectively (Smith et al., 2014). The cutthroat trout virus (CTV) is identified as an HEV-like virus in retrospective studies. CTV shares even lower sequence identity with mammalian and avian HEV and is now classified as a member of the genus *Piscihepevirus* (Batts et al., 2011; Smith et al., 2014).

For the four genotypes (1–4) of HEV infecting humans, there are differences in their geographic distributions. Genotype 1 HEV mainly includes strains from Asia and Africa including the Sar55 isolate, while genotype 2 contains a Mexican strain and variants from Africa. Genotypes 3, including human and swine HEV, is mainly found in the industrialized countries (Purcell and Emerson, 2008). The genotype 4 is previously thought to be found only in China (Purcell and Emerson, 2008), however, recent reports show that genotype 4 HEV strains are also isolated in other countries, including India, Indonesia, Japan, Vietnam, Spain, France, and Italy (Okamoto, 2007; Midgley et al., 2014; Lapa et al., 2015). For details about molecular epidemiology and viral evolution of HEV, please refer to the article by Purdy and Khudyakov (2011).

## Viral Proteins of HEV and Their Functions

### ORF1-Encoded Polyprotein

**FIGURE 2:**
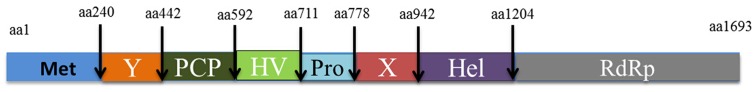
**Schematic illustration of putative domains in ORF1 polyprotein.** Met: Methyltransferase domain; Y: Y domain; PCP: papain-like cysteine protease; HV: hypervariable region; Pro: proline-rich domain; X: X-domain; Hel: helicase; RdRp: RNA-dependent RNA polymerase. The numbers above the box indicate amino acid residues of ORF1-encoded polyprotein of Sar55 strain.

The ORF1 is the largest ORF in the HEV genome and has 5082 nt in length according to the Sar55 strain (Tsarev et al., 1992; Emerson et al., 2001). It starts at the 5′ end of the genome after a 25 nt non-coding region and can be translated directly from the HEV genome. ORF1 encodes a 1693 amino acid (aa) polyprotein, which is needed for HEV replication. Bioinformatics analysis for the protein sequence encoded by ORF1 found eight putative domains according to their similarity to counterparts in the other viruses (Koonin et al., 1992). Moreover, the ORF1 sequence is highly related to the group of Rubi-like viruses including *Rubivirus*, *Betatetravirus*, *Benyvirus*, and *Omegatetravirus* (Koonin and Dolja, 1993; Liu et al., 2009). These functional domains include methyltransferase domain (Met), Y domain (Y), papain-like cysteine protease (PCP or PLP), hypervariable region (HVR), proline-rich region (Pro), X domain, helicase domain (Hel) and RdRp (**Figure 2**). In recent publications, the proline-rich region is frequently named together with HVR as the HVR.

The current data are conflicting about whether the HEV ORF1 product functions as a single polyprotein or needs to be further processed into smaller units by viral or cellular proteases (Ansari et al., 2000; Ropp et al., 2000; Sehgal et al., 2006; Suppiah et al., 2011; Perttila et al., 2013). One study using a vaccinia-derived expression system demonstrated that the ORF1 polyprotein could be cleaved by the PCP within it (Ropp et al., 2000). More than 10 years after that publication, the same group showed a lack of processing of the ORF1 polyprotein in HEK293T cells (Suppiah et al., 2011). Two fragments were found in another study on *in vitro* translation of full-length ORF1, but they were not observed in pulse-chase assay in human cells and their production was not dependent on the predicted protease domain in ORF1 product (Perttila et al., 2013). Furthermore, in *Escherichia coli* and a cell-free system based on HepG2 cells, ORF1 was expressed as a 186 kDa protein without further processing detected (Ansari et al., 2000).

On the other hand, other studies demonstrated contrasting results. Transfection of HepG2 cells with *in vitro* transcribed RNA from HEV cDNA produced cleaved products with sizes of 35, 38, and 36 kDa for the Met, Hel, and RdRp domains, respectively (Panda et al., 2000). Another study focusing on the analysis of the ORF1 functional domains also observed proteolytic processing of the HEV ORF1 fragment in insect cells (Magden et al., 2001). In a later study, the ORF1 product expressed in insect cells by baculovirus expression system was shown to exist as smaller fragments and this proteolytic processing could be inhibited by E-64d, a cell-permeable cysteine protease inhibitor (Sehgal et al., 2006). A recent publication reported that the refolded PCP domain expressed in *E. coli* is able to process ORF1 polyprotein *in vitro* (Paliwal et al., 2014). Moreover, based on an HEV-Sar55 replicon system in S10-3 cells (a subclone of Huh7 cells with improved HEV replication; Graff et al., 2006), the putative catalytic aa residues in the ORF1 protease domain are indispensable for HEV replication (Parvez, 2013). Overexpression of ORF1 from HEV Sar55 strain in S10-3 cells also resulted in cleaved products (Parvez, 2013).

Thus, despite the lack of conclusive data, the majority of studies so far are in favor of the polyprotein proteolysis. The cleaved ORF1 products could be possibly detected in the HEV-infected cells if effective and specific antibodies against the domains are available. A recent study employing yeast two hybrid (Y2H) demonstrated intraviral interactome within the domains from ORF1, further supporting the proteolysis of ORF1 polyproteins (Osterman et al., 2015). Moreover, ORF1 could be a determining factor for host tropism as a recombinant HEV harboring ORF1 of a genotype 4 HEV strain and the rest genome from genotype 1 strain replicates in transfected porcine kidney cells (Chatterjee et al., 2016). Therefore, ORF1 products involve in determination of HEV host tropism and should be further investigated. It is possible that proline rich region or HVR may be involved in host tropism determination since other domains are more conserved among the four HEV genotypes.

#### Met Domain

The Met domain is the first one at the N-terminus of the ORF1-encoded polyprotein. As the HEV genome is capped and the capping is crucial for its infectivity, a viral-specific methyltransferase was expected (Emerson et al., 2001; Zhang et al., 2001). Based on sequence analysis, the region in aa residues 60–240 was assumed to be a putative methyltransferase (Koonin et al., 1992). The HEV Met domain is similar to that of *Tricornaviruses*, which belong to the alpha-like supergroup of RNA viruses (van der Poel et al., 2001). There are an invariant His residue, an AspXXArg signature and an invariant Tyr residue in methyltransferase motifs I, II, and IV, respectively (Rozanov et al., 1992). Expression of HEV ORF1 cDNA (aa residues 1–979) in insect cells yields a 110 kDa protein (P110), along with a 80 kDa protein that is believed to be the proteolytic product of P110 (Magden et al., 2001). *In vitro* assays shows that the P110 possesses guanine-7-methyltransferase and guanylyl transferase activity (Magden et al., 2001).

#### Y Domain

The second domain after the methyltransferase is the Y domain, which is assumed to start from aa residue 216 and ends at aa 442. It is highly similar to that of the rubella virus (Koonin et al., 1992). Currently, there is no information available for the function of this Y domain in either HEV or the rubella virus.

#### PCP Domain

Papain cysteine protease domain is downstream of the Y domain. The PCP domain demonstrates moderate similarity to the protease domain in the rubella virus (Koonin et al., 1992). In the rubella virus, the PCP domain is responsible for the proteolytic processing of its NSP (Marr et al., 1994). Mutation of the catalytic residue within the PCP (Cys1152) abolishes its protease activity and results in inhibition of the NSP processing. It is also involved *in trans* and *cis* cleavage of the rubella virus NSP (Liang et al., 2000). However, regarding the function of the HEV PCP domain, the current data are incomplete and controversial.

In the vaccinia-mediated ORF1 expression system, mutation of the putative catalytic core (Cys483) of HEV PCP had no effect on proteolytic processing of the ORF1 product (Ropp et al., 2000). Another putative catalytic site of His590 in PCP is not conserved among different HEV strains. Later studies showed controversial data for the processing of the HEV ORF1 product (Ansari et al., 2000; Ropp et al., 2000; Sehgal et al., 2006; Suppiah et al., 2011). This leads to the speculation that whether HEV PCP is a real cysteine protease. Recently, Parvez demonstrated that the mutation of six cystine residues (C457A, C459A, C471A, C472A, C481A, C483A) and three histidine residues (H443L, H497L, H590L) in the PCP domain completely abolished HEV RNA replication in a Sar55-based replicon system in S10-3 cells. Notably, of these essential Cys and His residues, C483 and H590 were previously predicted as putative catalytic residues in the PCP domain (Parvez, 2013). Furthermore, the PCP domain expressed in the *E. coli* C43 strain (resistant to toxic protein expression) possesses protease activity (Paliwal et al., 2014). The purified protein cleaves both HEV ORF1 and ORF2 products that are *in vitro* translated. Protease inhibitor assay indicates the HEV PCP domain is a chymotrypsin-like protease (Paliwal et al., 2014). This observation suggests that HEV PCP is a real protease for HEV ORF1 polyprotein processing.

In recent years, the connection between ubiquitination and innate immunity signaling has been demonstrated (Zeng et al., 2009; Mao et al., 2010; Liu et al., 2013), and the antiviral function of some ubiquitin-like molecules, such as interferon-stimulated gene 15 (ISG15) and small ubiquitin-like modifier (SUMO), has been described (Liu et al., 2013). Some studies indicate that viral coded cysteine proteases possess deubiquitinase activity to inhibit host innate immunity, such as arterivirus papain-like protease 2 (van Kasteren et al., 2013) and PCP from porcine reproductive and respiratory syndrome virus (PRRSV; Li et al., 2010; Sun et al., 2010). Similar research performed on the HEV PCP domain suggests that it acts as an antagonist to ISG15 function to inhibit host innate immunity when expressed together with Met as the Met-PCP protein (Karpe and Lole, 2011). Moreover, study from our laboratory demonstrated that the PCP domain from HEV genotype 1 Sar55 strain is able to inhibit ubiquitination of RIG-I and TBK1, therefore resulting in the inhibition of RIG-I mediated signaling in innate immune responses (Nan et al., 2014b).

#### HVR Domain

Between the PCP domain and the X domain, there are HVR and Pro domains. These two regions were first named as HVR due to the extreme divergence in sequence between nt 2011 and 2325 (corresponding to residues aa 662–766) when the HEV Sar55 was compared with two other strains (Tsarev et al., 1992). In a later study, aa 712–778 in this region were designated a proline-rich region, which could be found in rubella virus as well. It was also considered to serve as a hinge between the X domain and its upstream domains because multiple proline residues in a protein or polypeptide may result in an unstable tertiary structure (Koonin et al., 1992; Tsai et al., 2001; Dosztanyi et al., 2006; Dunker et al., 2008). The length and sequence of HVR and Pro is highly variable among different HEV strains (Pudupakam et al., 2009; Smith et al., 2013).

Currently, there is some confusion regarding the nomen-clature of those two regions. Some of the recent publications designated the region of aa 712–778 as the hypervariable domain, which was originally referred to proline-rich region and left out the immediately upstream domain (aa 592–711; Pudupakam et al., 2009, 2011), whereas others still designate the aa 712–778 as the proline-rich region (Purdy, 2012). Current research mainly focuses on the Pro region and pays less attention to the upstream HVR domain. As a result, the function of HVR is unknown. However, data gained from the rubella virus shows that deleting part of the HVR domain along with part of the Pro region renders the mutant non-viable (Tzeng et al., 2001).

#### Pro Domain

The Pro domain is considered to be an intrinsically disordered region (IDR) with flexibility for insertion and deletion (Purdy, 2012; Purdy et al., 2012). Data from its counterpart in the rubella virus indicates that this region is not required for viral replication (Tzeng et al., 2001). As expected, deletion and mutation of this region in HEV indicates that it is not required for viral replication and infectivity, but it plays a role in replication efficiency *in vitro* (Pudupakam et al., 2009, 2011). It was also demonstrated that the Pro domain is interchangeable between genotypes with genotype-specific differences (Pudupakam et al., 2011). More interestingly, a remarkable HEV strain Kernow-C1, which was originally isolated from an HIV-positive patient with chronic HEV infection, contains an insertion of a 174 nt gene fragment of human ribosomal protein S17 in the Pro region (Shukla et al., 2011). This recombinant virus was adapted in culture cells and is able to propagate in cells from different species. It was speculated that insertion of the S17 fragment occurred in the host but was selected in cultured cells. This speculation needs further verification as direct detection of the inserted fragment from the host sample was not successful. Experimental insertion of the S17 fragment into the Pro domain of the Sar55 strain also generated a viable chimeric virus (Shukla et al., 2011). The S17 sequence insertion in HEV correlates with novel nuclear/nucleolar trafficking capabilities to the ORF1 protein of HEV Kernow C-1 P6 and the enhanced replication of this strain (Kenney and Meng, 2015a,b).

Although the Pro domain is considered highly diverse, some motifs are found in the IDR. Based on computer analysis and comparison with other IDRs, Purdy et al. identified several linear motifs (LMS), including two protease cleavage sites, three ligand binding sites and two kinase phosphorylation sites across all four genotypes (Purdy et al., 2012). The putative protein–protein interactions of the Pro domain were proposed in the same report as well, but need experimental verification. Nevertheless, this report provides some assumptions about the disorder-to-order state of the Pro domain. In another study, alignment of the Pro domain from different genotypes indicated the sequence is more conserved in genotypes 1 and 2 than genotypes 3 and 4 (Purdy, 2012). Adaptation to a wide host range for genotypes 3 and 4 is a possible reason. The authors also assessed the diversity of the Pro domain due to the higher rate of substitutions at the first and second codon positions, leading to a shift in translation to be more proline, alanine, serine, and threonine rather than histidine, phenylalanine, tryptophan, and tyrosine. This pattern matches the aa usage in proline-rich IDRs (Purdy, 2012). Furthermore, the C-terminus of this domain can tolerate more mutations than the N-terminus. Recently, the heterogeneity of the Pro and X domains is implicated in HEV persistence, which was revealed in an investigation into the association between the genetic heterogeneity of HEV quasispecies in ORF1 and the outcome of infection in solid-organ transplant patients (Lhomme et al., 2014).

#### X Domain

The X domain is located immediately downstream of the Pro domain. In HEV, its function is unknown. The HEV X domain homologs in other viruses such as rubella virus, alpha virus and coronavirus, are commonly identified as domain flanking the PCP domain (Gorbalenya et al., 1991; Koonin et al., 1992). It is also known as macro domain, due to its similarity with non-histone domain of the histone macroH2A. Macro domain has been identified in a variety of bacterial, archaeal, and eukaryotic organisms (Pehrson and Fried, 1992; Pehrson and Fuji, 1998).

Early studies of the human macro domain indicate that it is enriched in inactive mammalian X chromosomes, suggesting a role in gene silencing and inactivation (Costanzi and Pehrson, 1998). The macro domain inhibits transcription and binds to the transcription activator NF-κB (Perche et al., 2000; Angelov et al., 2003). Crystal structure analysis identifies a DNA binding motif in the macro domain, suggesting that it might interact with nucleic acids (Allen et al., 2003). A biochemical functional analysis indicates that the macro domain is involved in the downstream processing of ADP-ribose 1″-phosphate, a side product of cellular pre-tRNA splicing (Martzen et al., 1999). Furthermore, the macro domain is found in association with proteins involved in poly(ADP-ribose) polymerization, ADP-ribosylation and ATP-dependent chromatin remodeling (Aguiar et al., 2005).

Information about the function of viral macro domains is limited. ADP-ribose 1″-phosphatase activity has been demonstrated in the macro domain from coronavirus (Martzen et al., 1999; Putics et al., 2005, 2006; Saikatendu et al., 2005). Crystal structure analysis and *in vitro* assays on the macro domain of the SARS virus indicate that the viral macro domain has relatively poor ADP-ribose 1″-phosphohydrolase activity, but can bind free ADP-ribose and poly(ADP-ribose) efficiently (Egloff et al., 2006). In another report, the macro domains from Semliki Forest virus, HEV, SARS virus and yeast were compared with the human macro domain (Neuvonen and Ahola, 2009). The viral macro proteins bind poly (ADP-ribose) and poly (A), but have a low affinity for monomeric ADP-ribose. This implies that viral macro domains are functionally different from human homolog and may participate in cellular pathways involving RNA rather than ADP-ribose derivatives. However, a recent study shows that viral macro domains (HEV, coronavirus, and venezuelan equine encephalitis virus) can reverse protein ADP-ribosylation by acting on ADP-ribosylated substrates through the hydrolytic activity of their macro domains (Li et al., 2016). Furthermore, other studies indicate that the expression of viral macro domain in liver cells inhibits apoptosis since it is functionally related to poly(ADP-ribose) polymerase-1 (PARP-1; Allen et al., 2003; Chen et al., 2009), suggesting a role in apoptosis during viral infection. Recently, a highly conserved “glycine-triad” (Gly815-Gly816-Gly817) was identified downstream of the macro domain of HEV, which is homologous to the rubella virus protease-substrate (G1299-G1300-G1301; Parvez, 2013). Mutagenesis study indicates that G816V and G817V mutations in the macro domain are lethal for Sar55 replication in S10-3 cells. Further analysis identified the N-terminus residues Asn806, Asn809, His812, Gly815-Gly816-and Gly817 formed a potential catalytic-site homolog of Coronavirus ADP-ribose-1′-monophosphatase, which has essential role in viral replication (Parvez, 2015a). As mentioned above, a recent report suggests that the quasispecies heterogeneity in the macro domain might facilitate HEV persistence in solid-organ transplant patients (Lhomme et al., 2014). Study from our laboratory demonstrates that X domain of HEV Sar55 strain inhibits the phosphorylation of IRF3, which is a key transcription factor for type I IFN induction (Nan et al., 2014b). Moreover, except interacting with light chain subunit of human ferritin and inhibiting ferritin secretion, the X domain interacts with HEV Met and VP13 (Anang et al., 2016; Ojha and Lole, 2016).

#### Helicase Domain

The RNA helicase domain is downstream of the X domain. It is encoded by many positive-stranded RNA viruses and is essential for their replication (Kadare and Haenni, 1997). Helicases are motor proteins that are able to unwind nucleic acid strands by using energy from ATP hydrolysis (Kadare and Haenni, 1997). Helicases can be divided into six superfamilies (SF1-6; Singleton et al., 2007). RNA virus coded helicases are mainly classified into SF1 and SF2. Helicases SF1 and SF2 contain seven signature motifs (I, Ia, II, III, IV, V, and VI) that form the core of the enzyme (Kadare and Haenni, 1997). The HEV helicase belongs to helicase superfamily SF1 and is proposed to possess both NTPase and RNA unwinding activities (Koonin et al., 1992; Kadare and Haenni, 1997). *In vitro* experiments demonstrate that the HEV helicase purified from *E. coli* expression has both of the activities. It drives the hydrolysis of rNTPs but also dNTPs at a lower efficiency, as well as unwinds RNA duplexes with 5′ overhangs (Karpe and Lole, 2010a). RNA 5′-triphosphatase activity has also been observed in the HEV helicase domain, which is proposed to function along with methyltransferase for catalyzing RNA capping (Karpe and Lole, 2010b). Recently, a mutagenesis study on HEV helicase demonstrated that motifs I, IV, and VI are dispensable, while motifs I and III are crucial and unique for HEV helicase function (Mhaindarkar et al., 2014). A recent study shows that a V239A substitution in the helicase domain of a swine HEV strain is potentially associated with increased virulence (Ward et al., 2015). However, no human infection was reported to be associated with this strain.

#### RdRp Domain

The last domain of HEV ORF1 polyprotein is the RdRp. All positive-stranded RNA viruses code an RdRp, which is necessary for viral replication (O’Reilly and Kao, 1998). The RdRp from all positive-sense RNA viruses are classified into three large supergroups. All RdRp domains contain approximately 300 amino acid residues, with the central and C-terminal parts showing high similarity between each other (Koonin, 1991). RdRp from HEV belongs to supergroup III and has the highest similarity to the domains in rubella virus and beet necrotic yellow vein virus (BNYVV; Koonin et al., 1992). All eight conserved motifs can be found in HEV RdRp, including an Mg^2+^ binding sequence (GDD), which is essential for RdRp activity. The purified HEV RdRp is able to bind the 3′ end of HEV RNA, and needs two stem–loop structures at the 3′ end of the poly(A) stretch for this binding (Agrawal et al., 2001). Expression of the RdRp in mammalian cells as a GFP fusion protein indicates that it localizes in endoplasmic reticulum (ER), which could be a potential replication site for HEV (Rehman et al., 2008). Recent studies indicate that the emergence of G1634R mutation in the HEV RdRp is possibly due to ribavirin-induced mutagenesis and is associated with treatment failure of ribavirin monotherapy in solid-organ transplant patients (Debing et al., 2014b; Lhomme et al., 2015; Todt et al., 2016).

### The Capsid Protein Encoded by ORF2

**FIGURE 3:**
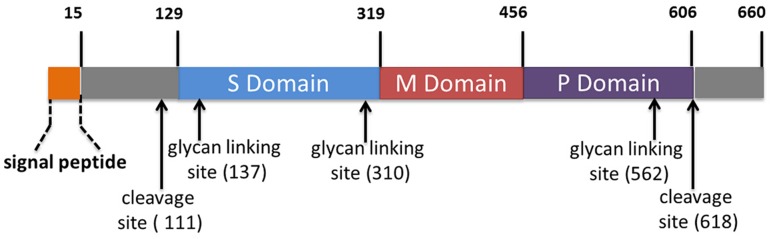
**Schematic illustration of the domains of the HEV capsid protein encoded by ORF2.** The numbers above the box and those in parentheses indicate amino acid residues of the capsid protein. S domain: shell domain; M domain: middle domain; P domain: protruding Domain.

The capsid protein is the major component of HEV virions. ORF2 is 1983 nt in length beginning from 37 nt downstream of ORF1 and ending at 65 nt upstream of the poly-A tail (Reyes et al., 1993; **Figure 1**). The deduced full-length ORF2 product has 660 aa residues with a predicted molecular mass of 72 kDa (Robinson et al., 1998). Recombinant ORF2 protein can bind to the 5′ region of HEV genome (Surjit et al., 2004). It was first shown that the ORF2 product exists as an 88 kDa protein, which carries N-terminal linked glycans and a potential ER-directing signal about 15 aa from its N terminus (Jameel et al., 1996). This 88 kDa protein can be further processed and has the potential to form non-covalent homodimers. A further study from the same group demonstrated Asn310 in ORF2 product to be the major site for glycosylation (Zafrullah et al., 1999; **Figure 3**). A mutagenesis study indicated that the N-terminal signal peptide is required for its cell surface expression via ER transition, but glycosylation of the capsid protein is not required (Zafrullah et al., 1999).

Since glycosylation of the capsid protein in non-enveloped viruses is not common, it is not known whether these modifications have biological significance for HEV infection. Mutations in the putative glycosylation sites (aa 137, aa 310, aa 562) prevent formation of viral particles and infection of rhesus macaques but without an effect on genome replication in cells (Graff et al., 2008). Mutation in the first two glycosylation sites prevents virion assembly, while mutation of the third site allows virion particle formation and RNA encapsulation (Graff et al., 2008). HEV particles released from cultured cells have a lipid component (quasi-enveloped), which can be removed by detergent treatment (Qi et al., 2015). The relationship between glycosylation of ORF2 and lipid envelope of viral particles needs to be clarified.

On the other hand, data acquired from studies using insect cells provide a different conclusion regarding ORF2 expression and processing. When expressed in insect cells, ORF2 product can be an insoluble full length protein of about 72 kDa, and a soluble form of 56.5 kDa, a processed product of the intact form (McAtee et al., 1996). Another group shows that when ORF2 is expressed in SF9 cells, a 62 kDa product is detected while lacking the first 111 aa residues of the putative ORF2 polypeptide (Zhang et al., 1997). Further studies in two different insect cell lines (SF9 and Tn5) show a soluble form of ORF2 product with a molecular mass of 50 kDa, which lacks the first 111 aa and the last 52 aa of ORF2 polypeptide but retains the ability to form virus-like particles (VLPs; Li et al., 1997, 2005d). VLP assembly is thought to involve dimer formation, and the C-terminus of the recombinant ORF2 protein is believed to be responsible for homo-oligomerization (Tyagi et al., 2001a; Xiaofang et al., 2001; Li et al., 2005a). A 3.5-Å resolution crystal structure obtained from HEV VLP indicates that the truncated HEV capsid protein has three domains designated as S (shell, aa 129–319), M (middle, aa 320–455), and P (protruding, aa 456–606; Yamashita et al., 2009; **Figure 3**). The VLP is composed of 60 subunits of the truncated capsid protein, forming icosahedral 2, 3, and 5-fold axes (Yamashita et al., 2009). Mutational analyses indicate that the protruding domain is involved in binding to the susceptible cells and contains neutralization epitopes (Yamashita et al., 2009). Moreover, the HEV VLP can be used as a delivery system to display foreign epitopes on its surface (Xing et al., 2011).

The ORF2 product expressed in insect cells is reactive with anti-HEV antibodies (Tsarev et al., 1993). Genetic analysis of ORF2 showed over 85% similarity among the four major HEV genotypes in mammalian hosts (Mori and Matsuura, 2011). Amino acid alignment indicates that divergences are mainly in the first 111 aa of the N terminus, which is not a component of the virions (Mori and Matsuura, 2011). A study manipulating a phage display system for overlapping peptides and truncated ORF2 proteins maps the major neutralizing domain to residues 458–607, which matches the location of the P domain (Schofield et al., 2000; Meng et al., 2001; Zhou et al., 2004). Both conformational and linear neutralizing epitopes have been identified from the HEV capsid protein (Gu et al., 2015; Tang et al., 2015).

These data provide valuable information for vaccine development. The ORF2 truncated proteins generated by baculovirus or bacterial expression systems have been tested in clinical trials (Shrestha et al., 2007; Zhu et al., 2010). However, a recent study that evaluated cross-protection against heterologous HEV indicates that vaccination of pigs with truncated capsid proteins derived from swine, rat and chicken HEV only elicits partial protection against a genotype 3 mammalian HEV (Sanford et al., 2012). Study of avian HEV capsid protein indicates that the N-terminal 338 aa residues react with swine and human anti-HEV sera (Wang et al., 2014b). Moreover, a recent study suggests that antigenic composition and immunoreactivity differed between HEV recombinant capsid proteins from different genotypes (Behloul et al., 2015), which raises the concerns about the efficacy of the current HEV subunit vaccine marketed in China. On the other hand, experiments based on the newly identified quasi-enveloped HEV particles indicate that lipid membrane protects the virions from neutralizing antibodies against the capsid protein (Yin et al., 2016).

Additionally, as a structural protein, the HEV capsid protein has been found to interact with some cellular proteins and plays a role in cell signaling. In one study, the capsid protein activates the pro-apoptotic gene CHOP, and increases the expression of HSP72, HSP70B and HSP40, and interacts with HSP72 (John et al., 2011). In addition, the capsid protein interacts with β-TRCP, a component of the ubiquitination complex that inhibits IκBα ubiquitination-mediated NF-κB activation (Surjit et al., 2012). However, these data are all based on overexpression of ORF2 in mammalian cells, and need to be further verified in whole virus infection.

### ORF3-Encoded Protein VP13

The ORF3 is the smallest among the three ORFs of HEV and overlaps with ORF2 by approximately 300 nt in a different reading frame. However, it does not overlap with ORF1 (Graff et al., 2006). The overlapping region with ORF2 (nt 5145–5475) was found to be the most conserved region between the Sar55 and BUR121 strains (Tsarev et al., 1992). An early study proposed that ORF3 encodes a protein with 123 aa and comes from a different subgenomic RNA other than that encoding ORF2 (Tam et al., 1991). However, a later study based on an HEV replicon shows that ORF3 is translated from the bicistronic subgenomic RNA and initiates at the third AUG of the presumed ORF3 at nt 5131 for Sar55 and the product is a protein with 113 or 114 aa and molecular size of 13 kDa (VP13), which is 9 aa shorter than the earlier predicted version (Graff et al., 2006). This observation has been confirmed by another study using a different HEV strain (Huang et al., 2007).

Sequence analysis has indicated that VP13 is unique and has no similarity to any other proteins known. It contains two hydrophobic domains in its N-terminal half and two proline-rich domains in its C-terminal portion (Kannan et al., 2009; Holla et al., 2013). A phosphorylation site (Ser71) was identified in the first proline-rich domain and can be phosphorylated by MAP kinase (Zafrullah et al., 1997). Furthermore, two PSAP motifs have been identified in genotype 3 VP13, with the first PSAP motif located at aa 86–89 and the second at aa 95–98, whereas genotypes 1, 2, and 4 have only one PSAP motif at aa 95–98 (Nagashima et al., 2011b). The second PSAP motif is needed for HEV virion release. Interestingly, among the four genotypes that infect humans, only genotype 1 vp13 has an additional proline-rich region that contains a PXXP motif in aa 66–75, which is linear and surface-oriented (Nan et al., 2014a,b, 2015). The unique motif reacts with a genotype 1 VP13-specific monoclonal antibody. This proline-rich region contains residues PMSPLR, a typical motif (PXXPX+) (+ is either arginine or lysine, X can be any aa) for class II SRC homology 3 (SH3) domains. SH3 domains are known to bind to proline-rich sequences containing a core PXXP motif flanked by a positively charged residue (Baumann et al., 1998; Raeder et al., 1998). SH3 domains comprise of about 60 residues and proteins containing SH3 domains typically play a role in signaling pathways involved in cell growth, differentiation and other regulatory functions (Zarrinpar et al., 2003). The next proline-rich region spanning aa 95–102 are RPSAPPLP, containing an additional residue than the typical motif (+XXPXXP) for class I SH3 domains. But interestingly only the PXXP motif in the second proline-rich region is known to interact with SH3 domains (Korkaya et al., 2001). The function of the PXXP motif in the first proline-rich region (aa 66–75) of VP13 of genotype 1 HEV is unknown. It might play a role in cellular signaling as proline-rich motifs are also involved in interacting with other domains besides SH3 (Zarrinpar et al., 2003).

Although the full function of HEV VP13 has not been defined yet, some studies have suggested that VP13 plays multiple roles during HEV infection. Early studies focusing on VP13 antigenicity and epitope mapping demonstrated that the last 32 aa of VP13 are an immunodominant region, and a synthesized peptide from that region is reactive with anti-HEV serum from a recovered patient (Semiletov et al., 1995; Dement’eva et al., 1997). However, another study mapping the T cell epitopes in ORF2 and ORF3 products indicated that no T cell proliferation was observed when cells were stimulated with peptides from VP13 (Aggarwal et al., 2007). A recent study based on genotype 4 HEV shows that continuous amino acid motif, VDLP, at the C-terminus of genotype 4 HEV VP13, is a core sequence of a VP13 epitope (Wang et al., 2015). Although VP13 is dispensable for viral replication in cultured cells (Emerson et al., 2006), it is indispensable for HEV infection *in vivo*, implying an important role for VP13 in host invasion (Graff et al., 2005; Huang et al., 2007).

A yeast two-hybrid system is employed to screen for the interaction partners for VP13. The VP13 can bind to inactive mitogen-activated protein kinase (MAPK) phosphatase and lead to activation of the MAPK (Kar-Roy et al., 2004), suggesting that VP13 can modulate host gene expression since MAPK is related to cell signaling and gene expression. Another study shows that VP13 inhibits the nuclear translocation of STAT3 and down-regulates STAT3-mediated gene expression, such as acute-phase response proteins (Chandra et al., 2008). The VP13 can also increase the expression of glycolytic pathway enzymes by increasing the phosphorylation and transactivation activity of p300/CBP (Moin et al., 2009). Furthermore, microarray analysis of Huh7 cells with VP13 expression suggests that liver-specific genes can be modulated, as VP13 is able to modulate the phosphorylation of hepatocyte nuclear factor 4 (Chandra et al., 2011).

The VP13 can also up-regulate mitochondrial voltage-dependent anion channel genes, which can protect cells from mitochondrial depolarization and death (Moin et al., 2007). This result implies that VP13 is able to inhibit the mitochondrial apoptosis pathway. The pro-survival role of VP13 is also demonstrated in another study showing VP13 delays the trafficking and degradation of the activated hepatocyte growth factor receptor to prolong endomembrane growth factor signaling (Chandra et al., 2010). Additional interacting molecules have been identified for VP13 by yeast two-hybrid screens, including α-1-microglobulin, bikunin, and bikunin precursor protein (AMBP), fibrinogen β chain and hemopexin (Tyagi et al., 2004, 2005; Ratra et al., 2008, 2009). Moreover, a recent study screening for intraviral protein interactions identified the Met, PCP, X, Helicase, and RdRp domains as interacting partners for VP13 as well (Osterman et al., 2015).

Besides yeast two-hybrid screens, overexpression of VP13 coding plasmid in mammalian cells was also employed to elucidate the function of VP13. The VP13 associates with the cytoskeleton fraction when expressed in cells, and deletion of the N-terminal hydrophobic domain of VP13 abolishes this association (Zafrullah et al., 1997). In a more detailed study, GFP-tagged VP13 is found to interact with microtubules to form a filamentous pattern in cells and modulate the microtubule dynamics (Kannan et al., 2009). VP13 leads to an elevation of acetylated α-tubulin, indicating increased microtubule stability (Kannan et al., 2009). Since there are two hydrophobic domains located in the N-terminus of VP13, truncation analysis indicated that both the hydrophobic domains are required for its association with the microtubules. Moreover, salt extraction studies have suggested that the VP13-microtubule interaction is electrostatic and motor protein dynein is needed for the interaction (Kannan et al., 2009). An earlier study showed that VP13 cannot be co-precipitated with tubulin by anti-tubulin antibody (Zafrullah et al., 1997). These results suggest that VP13 may associate with microtubules through interaction with another protein. This microtubule-like distribution of VP13 suggests that it may play a role in promoting virus egress, as the pUL37 protein of herpesvirus can interact with dystonin, an important cytoskeleton cross-linker involved in microtubule-based transport, in order to promote capsid transport on microtubules during egress (Pasdeloup et al., 2013). Besides cellular proteins, VP13 has been shown to interact with viral helicase, PCP and methytransferase from HEV ORF1, which suggests a regulatory function for VP13 in orchestrating the formation of the replicase complex (Osterman et al., 2015).

More interestingly, another study using monoclonal antibody against VP13 to capture HEV particles showed that VP13 can associate with virions and support virus release (Takahashi et al., 2008). The requirement of VP13 for virion release was later confirmed by a cell culture-adapted genotype 3 HEV strain with VP13 deletion (Yamada et al., 2009). Studies in Caco-2 cells and Huh7 cells for the Sar55, a genotype 1 HEV strain, showed that the intact PSAP motif spanning aa 96–99 in VP13 is required for virion release (Emerson et al., 2010; Nagashima et al., 2011b). For avian HEV, the PSAP motif in VP13 has also been found to play a role in virus release (Kenney et al., 2012). The PSAP motif in VP13 is required for the formation of membrane-associated HEV particles with the VP13 protein itself associated with lipids. This process is mediated by the cellular Tsg101 protein (Nagashima et al., 2011a,b). Replacement of the VP13 PSAP motif with heterologous late domain motifs (PPPY, YPDL, and PSAA) affects the virus release (Kenney et al., 2015). The specific interaction between VP13 and Tsg101 as well as involvement of endosomal sorting complex required for transport (ESCRT), which commonly participates in budding of many enveloped viruses, leads to the biogenesis of membrane-associated, “quasi-enveloped” HEV particles (Hurley, 2010; Feng et al., 2014; Nagashima et al., 2014; Yin et al., 2016). Therefore, VP13 is associated with virion during egress and anti-VP13 antibodies are able to capture HEV virions from the serum and cell culture supernatant, but not fecal samples from patients (Takahashi et al., 2008). A possible explanation is that viral particles could lose lipid-associated VP13 after passing through the gut (Takahashi et al., 2008). The role of VP13 in virus release may be one of its functions during HEV replication *in vivo*, indispensable for viral spread during infection.

On the other hand, as a small phosphorylated protein, VP13 can be phosphorylated at Ser71 by MAPK when expressed in COS1 and Huh7 cells (Zafrullah et al., 1997). A later study indicates that the Ser71 phosphorylation site is required for the interaction of the capsid protein and VP13 as VP13 can interact with the capsid protein in a yeast two-hybrid screen, especially for the non-glycosylated capsid protein (Tyagi et al., 2002). This finding also supports a role for VP13 in HEV structural assembly. However, a mutagenesis study shows that HEV lacking the phosphorylation site in VP13 is able to replicate its genome in cultured cells, and to infect rhesus monkeys similarly to wild type HEV in viremia and seroconversion (Graff et al., 2005). These data suggest that phosphorylation of VP13 is not necessary for genome replication or for the production of infectious virions. Moreover, in addition to phosphorylation and interaction with the capsid protein, VP13 can form a homodimer via the 43 aa domain located in the C-terminus (Tyagi et al., 2001b). On the other hand, VP13 has been reported to activate MAPK-JNK1/2 in hepatoma cells (Parvez and Al-Dosari, 2015).

Besides the functions mentioned above, VP13 also plays a role in the interferon induction and signaling. Data from our laboratory show that VP13 is able to enhance RIG-I activation, which leads to enhanced RIG-I signaling (Nan et al., 2014a). The VP13 extends RIG-I half-life and interacts with the N-terminal portion of RIG-I to enhance its activation by polyI:C. Interestingly, there is a genotype difference in the enhancement of RIG-I: genotypes 1 and 3 VP13 but not genotypes 2 and 4 VP13 have the role, implicating that VP13 may relate to HEV virulence and pathogenesis. On the other hand, another study demonstrate that VP13 of a genotype 3 HEV strain is able to interact with the STAT1 (signal transducer and activator of transcription) to inhibit interferon-α mediated signaling in A549 (human lung adenocarcinoma epithelial cell line; Dong et al., 2012).

In summary, as the product of the smallest ORF of HEV, VP13 has multiple functions and plays an indispensable role in infectivity in experimentally infected animal models. However, it is not required for HEV replication in cultured cells. Our current knowledge indicates that VP13 is a multifunctional protein in interacting with many cellular proteins, modulating host gene expression and involved in virion release.

### Novel ORF4 from Genotype 1 HEV

Recently, a novel ORF4 (nt 2835–3308) was identified from genotype 1 HEV (Nair et al., 2016). Unlike other ORFs in HEV, translation of ORF4 is driven by an IRES-like sequence located in nt 2701–2787 of HEV genome (Nair et al., 2016). The ORF4 product interacts with multiple viral proteins to form a protein complex consisting of viral RdRp, helicase and X, and the ORF4 product stimulated viral RdRp activity to promote viral replication. Expression of the ORF4 was verified in a cell-free system and antibodies against this protein were determined from HEV-infected patients (Nair et al., 2016). However, analysis of HEV sequences from other genotypes suggests ORF4 is not conserved across genotypes (Nair et al., 2016). Therefore, more investigation is needed to elucidate the exact function of ORF4.

## Replication Cycle of HEV

Due to the lack of an effective *in vitro* cell culture system for HEV, the replication cycle of HEV is largely unknown. The capsid protein is believed to bind to an unidentified cellular receptor to initiate viral entry. HEV-VLPs generated from recombinant ORF2 protein attach to cells via heparin sulfate proteoglycans (HSPGs; Kalia et al., 2009). Moreover, one study based on a viral overlay protein binding assay (VOPBA) suggests that a protein with molecular weight about 55 kDa could be the candidate receptor for HEV entry; but mass spectrometry revealed that this virus binding band contained 31 different proteins (Zhang et al., 2011). Another study suggests that aa 458–607 located in the C-terminal region of the capsid protein (M domain) may be the putative receptor binding site of HEV virions (He et al., 2008). Moreover, structure and sequence analyses suggest that the putative binding motif of the capsid protein is conserved among all four major mammalian HEV genotypes (Guu et al., 2009). Heat shock cognate protein 70 (HSC70), HSPGs and Grp78 are found to be involved in either cell surface binding with HEV capsids or intra-cellular transport in different models and are potential cellular receptors or essential factors for HEV proliferation (Kalia et al., 2009; Yu et al., 2011; Cao and Meng, 2012). However, further investigation is needed to confirm if these molecules truly act as receptors for HEV. After binding with its receptor, HEV particles are internalized via a dynamin-2, clathrin, and membrane cholesterol-dependent pathway (Kapur et al., 2012; Holla et al., 2015). In addition, a recent report suggests the quasi-enveloped HEV particles enter cells via a distinct pathway that involves in degradation of the lipid membrane in the lysosome (Yin et al., 2016).

After entry into permissive cells, the HEV capsid is uncoated by unknown mechanisms. In one study utilizing VLP from the truncated capsid protein HEV239, an HSP90-specific inhibitor (geldanamycin) blocks the intracellular transport of the HEV239 VLP without affecting its entry (Zheng et al., 2010). This suggests that HSP90 may play a role in the intracellular transport of HEV particles. After uncoating, the HEV ORF1 translation is followed. HEV genomic RNA replication relies on the replicase encoded by ORF1. Along with the generation of the sub-genomic RNA, translation of ORF2 and ORF3 occurs, followed by virion packing and egress. For the release of HEV particles, multivesicular body (MVB) pathway and ESCRT machinery in the cytoplasm are used (Nagashima et al., 2014).

## Cell Culture Systems and Propagation of HEV

Since the discovery of HEV, many efforts have been made to develop a rigorous *in vitro* cell culture system. However, the cell culture system of HEV is still limited and relatively ineffective, especially for genotype 1 HEV. An early study tried to use primary hepatocytes from macaques with serum-free medium for HEV propagation; however, HEV replication was limited and the detection of HEV in the medium relied on PCR amplification (Tam et al., 1996). A group from Japan reports that HEV isolate 87A is able to replicate in A549 cells; however, PCR was also used to detect viral RNA in the cell culture supernatant (Huang et al., 1995), instead of immunofluorescence assay to detect viral proteins. Another group also showed that the A549 cell line could be used effectively for passaging two Chinese HEV isolates (Wei et al., 2000).

On the other hand, as the commonly employed method for single-stranded, positive-sense RNA virus, transfection of capped RNA from an HEV cDNA infectious clone via *in vitro* transcription to PLC/PRF/5 (hepatocellular carcinoma) and Huh7 cells demonstrates limited replication of HEV (Emerson et al., 2004). Although cell lysates from the RNA transfected cells is infectious in rhesus monkeys, cell to cell spread of the virus in cultured cells is not observed (Emerson et al., 2004). S10-3 cell line, a subclone of Huh7 hepatoma cell line, has improved replication efficiency of HEV for the Sar55 strain (Graff et al., 2006; Shukla et al., 2011). But this assay still relies on transfection of the cells with full length HEV RNA. A recent report suggests that replication efficiency of genotype 1 HEV in human hepatoma cell lines (Huh7, Huh7.5, and HepG2/C3A) is affected by innate immune response (Devhare et al., 2016).

A Japanese group reports that a genotype 3 isolate from acute hepatitis patient propagates in PLC/PRF/5 and A549 cells (Tanaka et al., 2007; Okamoto, 2011). After A549 cells were seeded in a six-well plate and inoculated with HEV at 1.0 × 10^4^ and 1.0 × 10^5^ RNA copies per well, HEV RNA reached the highest titer of 10^7^ copies/ml at 50 days post-inoculation. However, PLC/PRF/5 cells could only support efficient growth as A549 with a higher MOI (1.0 × 10^5^ viral RNA copies per well). Moreover, in this HEV cell culture system, HEV infected cells need to be maintained at 35.5°C and cultured with a mixed cell culture medium (50% Dulbecco’s Modified Eagle Medium and 50% Medium 199) supplemented with 2% (v/v) fetal bovine serum and 30 mM MgCl_2_. The same group also reports that a genotype 4 HEV from a fulminant hepatitis patient can grow in PLC/PRF/5 and A549 cells and reach a titer of 1.3 × 10^7^ copies/ml within 10–20 days incubation period (Tanaka et al., 2009). Moreover, a human hepatoma-derived cell line HepaRG and a porcine embryonic stem cell-derived cell line PICM-19, which have morphological and functional properties similar to primary hepatocytes, were shown to support HEV replication (Rogee et al., 2013). However, the HEV replication level in these two cell lines is very low and requires 1 month incubation (Rogee et al., 2013).

In HEV Kernow-C1 p6 cell culture system, the recombinant virus with human S17 gene insertion was presumed as a minor species in the host but was selectively adapted to cells after six passages (Shukla et al., 2011). Replication of Kernow-C1 p6 is 7.5-fold higher in HepG2/C3A human hepatoma cells than in Huh7.5, PLC/PRF/5, A549, Caco-2 or rhesus kidney cells in a 7-day incubation period, suggesting that HepG2/C3A cell line is the most permissive. Moreover, this HEV isolate is also able to infect a variety of non-primate cells, including cow, mouse, chicken, cat, dog, and rabbit cells, albeit with lower efficiency. Although it is still unclear how the insertion of S17 occurred in HEV infected patient, Okamoto’s group demonstrated that two cell adapted HEV strains HEV JE03-1760F (genotype 3) and HEV JF5/15F (genotype 4) did not shown any recombination with cellular S17 gene after 53 and 33 generations of passages in PLC/PRF/5 and A549 cells, respectively (Okamoto, 2013), which suggested the recombination and insertion of S17 may occur in patients rather than in cultured cell. Besides human HEV isolates, animal HEV strains from domestic pigs, wild boars, rabbits, and rats can be propagated in human hepatoma cell lines as well (Jirintai et al., 2012, 2014; Takahashi et al., 2012). A recent report demonstrates that pluripotent stem cell derived hepatocytes support HEV replication *in vitro* (Helsen et al., 2016).

In summary, the current cell culture systems for HEV have limitations. So far, only one report shows limited replication of a genotype 1 HEV strain from serum sample in cell culture without RNA transfection (Takahashi et al., 2010). On the other hand, although several groups have demonstrated that genotypes 3 or 4 HEV strains can be adapted to cultured cells and are able to re-infect new cells, a long incubation time is needed in comparison to other RNA viruses with good cell culture systems. Moreover, the cell culture adapted Kernow-C1 virus may have a different phenotype compared with its parental wild type virus as this cell culture adapted virus contains host gene sequence.

## Epidemiology of HEV

The HEV is primarily transmitted via fecal-oral route. The most common source of infection is contaminated drinking water in developing countries. For a long time, hepatitis E was thought to be a public health problem only for developing countries. However, hepatitis E is now frequently recognized in industrialized countries where it was not thought to be endemic previously (Kwo et al., 1997; Erker et al., 1999; Schlauder et al., 1999; Worm et al., 2000; Kabrane-Lazizi et al., 2001; Mizuo et al., 2002; Sadler et al., 2006).

World Health Organization estimates that there are 20 million HEV infections annually across the world. Among these cases, there are over 3 million symptomatic cases and 56,600 deaths (WHO, 2015). Hepatitis E is highly endemic in East and South Asia. Data indicates over 50% of global hepatitis E deaths occur in this region. In East Asia, large outbreaks of hepatitis E have only been described in China. Hepatitis E accounts for 20–50% of acute hepatitis cases in this region. The seroprevalence of anti-HEV antibodies in the region varies from 10 to 50%, indicating that hepatitis E is hyperendemic in this region. In South Asia, outbreaks of hepatitis E have been reported in most countries in this region, but variable in scale (WHO, 2015). HEV accounts for 20–60% of sporadic acute hepatitis and fulminant liver failure in this region. In particular, the rates of fulminant liver failure are usually higher in pregnant patients. A recent paper reported that HEV infection causes 49% acute viral hepatitis and 75% fulminant hepatic failure in pregnant women in one area in India (Kumar et al., 2014). However, the seroprevalence rates of prior exposure to HEV are relatively low, ranging from 10 to 40% in most studies.

In the developed countries, such as North America, Western Europe and Japan, no outbreaks have been reported. These areas are considered as low or non-endemic for HEV. However, sporadic cases of hepatitis E have been reported. Transmission of HEV from animal reservoirs to humans is assumed to be the major cause of those sporadic cases. A series of cases of HEV infection in people who ate undercooked deer meat 6–7 weeks before the onset of disease have been reported (Tei et al., 2003; Yazaki et al., 2003; Li et al., 2005c). HEV RNA recovered from the leftover deer meat was found to be identical in sequence to the HEV RNA recovered from the patients (Takahashi et al., 2004). Consumption of shellfish is considered a risk factor in a documented case (Koizumi et al., 2004). Thus, foodborne infection may occur from the consumption of uncooked/undercooked products from infected animals. Moreover, blood transfusion and solid organ transplant mediated HEV transmission are reported (Pas et al., 2012; Wedemeyer et al., 2012; Sue et al., 2016). IgM and IgG against HEV are detected in recipients of blood transfusions (Wedemeyer et al., 2012).

Hepatitis E virus genotype 1 is responsible for most endemic and epidemic cases of hepatitis E in Asia, and genotype 2 is prevalent in Central America and Africa (Purcell and Emerson, 2008). There is no known animal reservoir for HEV genotypes 1 and 2 (Wedemeyer et al., 2012). Genotypes 3 and 4 are zoonotic and can cause HEV infections in the developed countries. For the detailed geographical distribution of hepatitis E virus genotypes, please refer to these reviews (Dalton et al., 2008; Kamar et al., 2012a).

## Pathogenesis, Clinical Signs, and Diagnosis of HEV Infection

Hepatitis E virus infection mainly causes acute hepatitis with a case fatality rate from 0.5 to 3% in young adults (Jameel, 1999). Remarkably, case fatality rate resulting from HEV-related fulminant liver failure can reach up to 30% in infected pregnant women in their third trimester of gestation (Jameel, 1999). Generally, HEV has an incubation period of 2–8 weeks (Purcell and Emerson, 2008). The initial symptoms of acute hepatitis E are unspecific and flu-like, such as myalgia, arthralgia, and weakness. After this short prodromal phase, a period of symptoms such as vomiting, itching, uncolored stools, darkened urine and jaundice could last for days to several weeks accompanied by increased levels of liver transaminases, bilirubin, alkaline phosphatase, and γ-glutamyltransferase (Hoofnagle et al., 2012; Wedemeyer et al., 2012). Current case reports indicate that most cases are self-limited and do not result in chronic hepatitis (Hoofnagle et al., 2012). An investigation on pregnancy outcomes in hepatitis E shows higher HEV loads in pregnant women with acute viral hepatitis and fulminant hepatic failure, and higher levels of TNF-α, IL-6, IFN-γ, and TGF-β1 than non-pregnant women, which suggests that high cytokine levels are correlated with severe liver injury in HEV infection (Kumar et al., 2014). A recent study highlights the role of TLR3 and IFN-γ in HEV pathogenesis. Patients with high levels of TLR3 and robust IFN-γ response are observed in self-limiting acute viral hepatitis cases, and are able to limit the disease and recover uneventfully (Majumdar et al., 2015). However, patients with lower expression of TLR3 and IFN-γ progress to acute liver failure (Majumdar et al., 2015).

HEV can cause chronic infection as well. Although chronic HEV infection was initially reported only in immuno compromised persons, such as organ transplant recipients, patients receiving cancer chemotherapy and HIV-infected persons (Hoofnagle et al., 2012), latest reports show that chronic HEV infection also occurs in an immunocompetent individual with systemic lupus erythematosus (SLE; Grewal et al., 2014). However, since this kind of cases are rare, data available so far are not sufficient to consider this patient as an immunocompetent individual (Kamar and Izopet, 2014). In organ transplant recipients, the chronic course leads to persistent increases in levels of alanine aminotransferase, significant histological activity and fibrosis in some cases (Wedemeyer et al., 2012). HIV-infected individuals have higher positive rate of anti-HEV antibody than individuals without HIV infection (Wedemeyer et al., 2012).

Besides hepatitis, extrahepatic manifestations have been documented. Neurological disorders, such as polyradiculopathy, Guillain–Barré syndrome, bilateral brachial neuritis, encephalitis and proximal myopathy, and neuralgic amyotrophy are reported in patients with acute and chronic HEV infections (Kamar et al., 2011, 2014; van den Berg et al., 2014; van Eijk et al., 2014; Dalton et al., 2016; Drave et al., 2016). The kidney injury caused by HEV infection is reported and also documented in monkeys infected experimentally with HEV as well (Kamar et al., 2005, 2012b; Geng et al., 2016). Furthermore, a recent report provides evidence that extrahepatic replication of HEV in the placenta of infected mothers, which may be associated with fetal mortality (Bose et al., 2014). It also raises the concern for the vertical transmission of HEV to fetus and newborn by an infected mother (Krain et al., 2014).

A report suggests the association between the outcome of HEV infection in solid-organ transplant patients and the genetic heterogeneity of HEV quasispecies in ORF1 (Lhomme et al., 2014). Analysis of the viral genetic heterogeneity indicates that both nucleotide complexity and genetic distance of the ORF1 proline-rich domain in patients whose infection became chronic are higher than the patients who cleared the virus (Lhomme et al., 2014).

Although diagnostic tests for HEV are commercially available, none of them have been formally approved in the United States by the Food and Drug Administration (FDA; Hoofnagle et al., 2012). Current tests mainly target anti-HEV antibodies, including IgG and IgM. However, several assays are based on antigens expressed by a single HEV genotype, especially genotype 3, and might be limited for the detection of all HEV genotypes. Indeed, there are variations in sensitivity, specificity and agreement in the results of these assays, which may account for the discrepancies among positive rates of anti-HEV antibodies in various populations (Mast et al., 1998; Herremans et al., 2007; Drobeniuc et al., 2010). It is also notable that a recent study demonstrates the false positive result in HEV IgM test due to cross reaction with EBV and CMV, which heavily affects the accuracy of HEV serology testing (Hyams et al., 2014). Only 13.3% of the total samples with the positive HEV IgM were PCR positive for HEV RNA. The cross reactivity of IgM against HEV, EBV, and CMV is very high. These data suggest that to confirm HEV infection in patients, clinical features, blood ALT level and PCR testing should be all included in addition to serological test alone.

On the other hand, although HEV RNA can also be detected in blood and stool for several weeks after acute HEV infection, in addition to a narrow detectable window of HEV viremia (Hyams et al., 2014), current HEV RNA tests are still experimental since they have not been standardized yet (Wedemeyer et al., 2012). Furthermore, diagnostic support for IgM and IgG anti-HEV detection in clinical samples using commercially available kits and PCR assay for detection of HEV RNA in serum and stool samples are also available from the Division of Viral Hepatitis in the Centers for Disease Control and Prevention (CDC, 2016).

## Treatment and Prevention of HEV Infection

Hepatitis E virus infection mainly causes a self-limited disease and most infected individuals are able to clear it spontaneously. Although the case fatality rate in adults is 0.5–3%, the rate can increase to 30% in pregnant women during their third trimester of gestation in South Asia (Jameel, 1999). Therefore, antiviral therapy is needed. Although no specific treatment has been approved for HEV, off-label application of ribavirin as monotherapy for HEV has demonstrated promising results in both acute and chronic hepatitis E patients (Kamar et al., 2010; Mallet et al., 2010; Gerolami et al., 2011). *In vitro* assay showed that ribavirin could inhibit replication of genotypes 1–3 HEV through the depletion of intracellular GTP pools in HEV infected cells (Debing et al., 2014a). For immunosuppressed patients, a reduction of immunosuppression has shown efficacy in the treatment of chronic HEV infection (Wedemeyer et al., 2012). Moreover, application of pegylated interferon in combination with ribavirin has been reported as a treatment for chronic HEV infection but only shown moderately synergistic effect (Wedemeyer et al., 2012; Debing et al., 2014a). However, due to the evidence of embryolethality and teratogenicity revealed by animal study, ribavirin has been assigned to pregnancy category X by the FDA and contraindicated in women who are pregnant and in the male partners of women who are pregnant (Sayed et al., 2015). Moreover, ribavirin-induced G1634R mutation was reported and associated with treatment failure of ribavirin monotherapy in solid-organ transplant patients (Debing et al., 2014b; Lhomme et al., 2015; Todt et al., 2016). Therefore, viral specific treatment for HEV is needed.

Our laboratory has successfully tested application of peptide-conjugated morpholino oligomers (PPMOs) as novel anti-HEV compounds (Nan et al., 2015). PPMOs are water soluble, nuclease-resistant single-stranded DNA analogs containing a backbone of morpholine rings and phosphorodiamidate linkages along with conjugation of arginine-rich cell penetrating peptide for facilitating cell delivery (Summerton, 1999; Abes et al., 2006). PPMOs bind to mRNA by Watson–Crick base pairing and interfere with translation through steric blockade of the AUG-translation initiating region. Antisense morpholino oligomers are currently tested in clinical trials for treating Duchenne muscular dystrophy in humans and has been documented as effective against numerous types of viral infections in experimental animal models (Anthony et al., 2012; Mendell et al., 2013; Moulton, 2013). Importantly, upon systemic administration, PPMOs distribute to liver, remain pharmacologically viable, and are effective at reducing viral titers (Amantana et al., 2007; Burrer et al., 2007; Paessler et al., 2008). In our study, PPMO HP1 targeting 5′ UTR of HEV genotype 1 Sar55 strain demonstrates strong inhibition of HEV replication (Nan et al., 2015). Since the 5′ UTR of HEV genome is highly conserved among different HEV genotypes infecting humans, the PPMO HP1 may be an HEV-specific inhibitor with antiviral activity across multiple HEV genotypes (Nan et al., 2015). These qualities, along with the *in vitro* efficacy against HEV (Nan et al., 2015), make PPMOs be appealing for consideration as a novel inhibitor of HEV infections.

Another nucleic-acid based strategy, siRNA, has also been reported to be effective in inhibiting HEV replication. An siRNA targeting HEV RdRp was reported to inhibit HEV replication in A549 cells and in piglets (Huang et al., 2009). In another report, siRNA targeting a 3′ *cis*-acting element and viral nucleotide sequences coding for helicase and RdRp are effective against HEV in HepG2 cells (Kumar et al., 2010). However, it is generally acknowledged that siRNA needs considerable improvements in their delivery to relevant targets *in vivo* before they can be considered for clinical applications involving systemic delivery against virus infections.

Current prevention for HEV relies on sanitary measures, such as providing clean water, and appropriately cooked food to avoid transmission from undercooked food (Kamar et al., 2012a). Since *in vitro* culturing of HEV is limited and ineffective, HEV vaccine development mainly focuses on the expression of the capsid protein as a subunit vaccine. The capsid protein shares over 85% identity among the four major HEV genotypes in mammalian hosts (Mori and Matsuura, 2011). The capsid protein from genotype 1 HEV expressed by baculovirus or bacterial vectors has been tested in clinical trials. The first candidate was a 56 kDa protein expressed in insect cells. In a phase 2 trial in Nepal, the vaccine is well-tolerated and highly immunogenic, with 95% efficacy for protection against hepatitis E (Shrestha et al., 2007). The second vaccine, HEV239, encompasses aa 368–606 of ORF2 product, is a 26 kDa truncated protein expressed in *E. coli* (Li et al., 2005b). This vaccine is well-tolerated with an efficacy of 100% protection after three doses in a population tested in China, which included both men and women aged 16–65 years (Zhu et al., 2010). The HEV239 vaccine was approved and marketed in China in 2012. Whether it will be endorsed in other countries or how effective it is against all other genotypes of HEV infecting humans remains unknown. Moreover, a study shows that HEV239 vaccine could protect rabbits against homologous and heterologous HEV challenge (Liu et al., 2014; Zhang et al., 2015). A recent report demonstrates that a hybrid protein fusing protruding (P) domains from capsid proteins of both Norovirus (NoV) and HEV induces a higher antibody titer than either P domain alone (Wang et al., 2014a). Subunit vaccine candidates containing antigens of HEV, rotavirus, and astrovirus are reported as well (Xia et al., 2016).

## Conclusion and Perspectives

More than 20 years have passed since the discovery and complete genome sequencing of HEV. Our understanding of HEV is still limited, though ongoing research continues to reveal more and more information about this virus. Currently, we know that HEV is not only a public health concern in developing countries as previously thought, but also a concern with a more complicated scenario in the developed countries. More and more animal reservoirs are revealed and we now understand that genotypes 3 and 4 HEV are zoonotic and foodborne pathogens. However, the cross-species transmission and host tropism of different HEV genotypes are still elusive. Current data imply certain viral proteins such as ORF1 product plays a role in the host tropism of HEV. Further investigation is needed to elucidate the basic biology of HEV.

On the one hand, although approved in China, the HEV239 vaccine is still unavailable to most of the world, despite the fact that serum surveillance indicates a high prevalence rate of HEV throughout the world. Moreover, recent discoveries about the antigenicity variation between HEV genotypes and quasi-enveloped viral particles hidden from neutralizing antibody suggest new challenges and questions about the efficacy of the approved vaccine. Further investigation about the vaccine efficacy against multiple HEV genotypes or seeking for an improved vaccine is needed. In addition, virus specific treatment for HEV infection is not available yet. Although, the off-label using of pegylated IFNs and antiviral drugs for general purposes have demonstrated efficacy against HEV, safety is still a concern as no validation has yet been conducted for these treatments. Therefore, a HEV-specific treatment such as PPMOs is needed.

Due to the absence of a suitable animal model and a simple cell culture system, many details about this virus and its infection, such as its biology, pathogenesis, strain variances, genotype differences, molecular mechanisms and vaccine efficacy for cross protection are still incomplete. However, current information also indicates that it is possible to establish a useful cell culture system using certain HEV strains such as the cell culture-adapted Kernow-C1 strain. These recent advances will facilitate further studies, which hopefully will reveal more insights about the basic biology of HEV, such as proteolytic processing of ORF1 product, functions of the viral proteins, HEV pathogenesis, effective therapeutics and a better vaccine.

## Author Contribution

All authors listed, have made substantial, direct and intellectual contribution to the work, and approved it for publication.

## Conflict of Interest Statement

The authors declare that the research was conducted in the absence of any commercial or financial relationships that could be construed as a potential conflict of interest.
